# Novel mutation in YMDD motif and direct neighbourhood in a child with chronic HBV-infection and clinical lamivudine and adefovir resistance - a scholarly case

**DOI:** 10.1186/1743-422X-7-167

**Published:** 2010-07-21

**Authors:** Verena Schildgen, Susanne Ziegler, Ramona L Tillmann, Oliver Schildgen

**Affiliations:** 1 Institute for Virology, Helmholtz Zentrum Munich, Munich, Germany; 2 Institute for Virology, University Hospital Essen, Essen, Germany; 3 Institute for Virology, University Hospital Bonn, Bonn, Germany; 4 Institut für Pathologie, Kliniken der Stadt Köln gGmbH/University Hospital Witten-Herdecke, Cologne, Germany

## Abstract

**Context:**

Chronic HBV infection is a major cause of hepatocellular carcinoma (HCC) which meanwhile has become the 5^th ^most reason for a fatal outcome of cancer. Worldwide, approximately 350 million people are chronically HBV infected and as such of risk to develop HCC, of those an estimated high rate of children. Treatment of chronic infection is sufficient to reduce the rate of HCC but the rate of sustained virological response remains to low, not at least due to emergence of resistant virus strains. Less is known on HBV infection in children despite the extremely high rate of chronicity.

**Objective, Design, Setting, and Patient:**

The case of a nine years old male with a 6 year history of chronic HBV infection, of those 5 years with antiviral treatment is described.

**Interventions and Main Outcome Measure(s):**

Before our lab was consulted, the patient was unsuccessfully treated with interferon, an obscure drug named Hepon, which should activate antiviral immune response, and Lamivudine, the latter most likely becoming ineffective due to the mergence of resistant subpopulations (rtL180 M, rtV207 M, two strains with stop codons at position rt188 and rt198, rtM204V (YVDD), rtM204K (YKDD)). Replacement of Lamivudine by adefovir displayed no advantage despite the lack of resistance mutations, thus no decrease in viremia was observed under adefovir treatment.

**Results and Conclusions:**

Novel mutations in the YMDD motif and its direct neighbourhood were observed, both being compatible with Lamivudine resistance. No mutations were found that are associated with ADF resistance. Both, the clinical course of treatment and the genotypic resistance profile emphasize the need for systematic analyses of the HBV resistance mechanisms and structured therapy concept also for children chronically infected with HBV.

## Introduction

Hepatitis B virus (HBV), discovered in 1966, is one of the major serious and global public health problems affecting approximately 2 billion people worldwide http://www.who.org. Estimated 350 million persons are chronically infected with HBV. Approximately 15-40% of infected patients will develop cirrhosis, liver failure or hepatocellular carcinoma (HCC) [1]. HCC incidence has increased worldwide to the 5^th ^most frequent cancer killing 300,000 - 500,000 people each year.

The estimated worldwide mortality caused by HBV infection is 0.5-1.2 million deaths per year, although safe and effective vaccines against HBV infections have been available since 1982 [2]. Furthermore, the approval of oral antiviral agents has revolutionised HBV treatment since 1998, and enabled effective clinical management of the disease. Acute hepatitis B in adults is self-limiting in most cases (95% of adults), therefore antiviral therapy is indicated only for patients with protracted severe acute hepatitis or fulminant hepatitis [3,4]. Although this scheme should be applied also for pediatric patient cohorts less is known on the treatment of chronically HBV infected children. Also in those patients two major strategies are believed to have potential to treat chronic HBV infection in adults, namely direct interference with viral replication and modulation of the host's immune responses, the latter known to be immature in pediatric patients and thus resulting in high chronicity rates.

The lack of knowledge in this cohort may be a result of lacking clinical studies, the lack of drugs approved for the treatment of chronically HBV infected children, and the fact that the majority of chronically HBV infected children comes from poor countries never having access to antiviral therapy. Here we report the case of a young male patient suffering from chronic HBV who after non-response to interferon and initial treatment with lamivudine developed resistance associated with YVDD and a novel mutation in the YMDD motif. Furthermore, the patient showed initial non-response to adefovir despite the lack of resistance mutations, giving raise to the hypothesis that host factors influenced this treatment failure.

## Case Report/Clinical Data

A male patient adopted from Cameroon, 4-year-old at the beginning of the therapy history, was chronically infected with Hepatitis B virus infection perinatally, as assumed from anamnestic investigations. Chronic Hepatitis B virus infection was diagnosed at the age of 3. Written informed consent for publication of the clinical course was obtained by the legal representatives of the patient.

**Figure 1:**
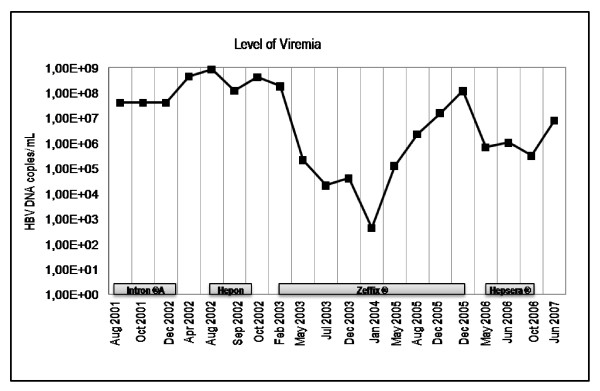
**Level of viremia in a patient receiving Interferon, Hepon, Lamivudine and Adefovir**. The levels of viremia were measured by PCR-based amplification. The limit of detection of each assay is 0.17 × 10^3 ^copies per mL.

First, he had undergone unsuccessful therapy with Intron^® ^A (recombinant interferon alpha-2b, 3 × injections per week) for nine month, leading to severe side effects, including fever, insomnia with disorientation and ostealgia. Within the first seven month of IFN-treatment, the level of viremia increased from 4.0 × 10^7 ^molecules of HBV DNA per milliliter to 8.4 × 10^8 ^molecules of HBV DNA per milliliter (figure 1). Interferon therapy was replaced by Hepon application (2 × daily sublingual applications). Hepon is suspected to be an immunmodulator with antiviral activity [5], but clinical data and studies are missing and the drug is not approved. The level of viremia did not change significantly upon Hepon treatment.

**Figure 2:**
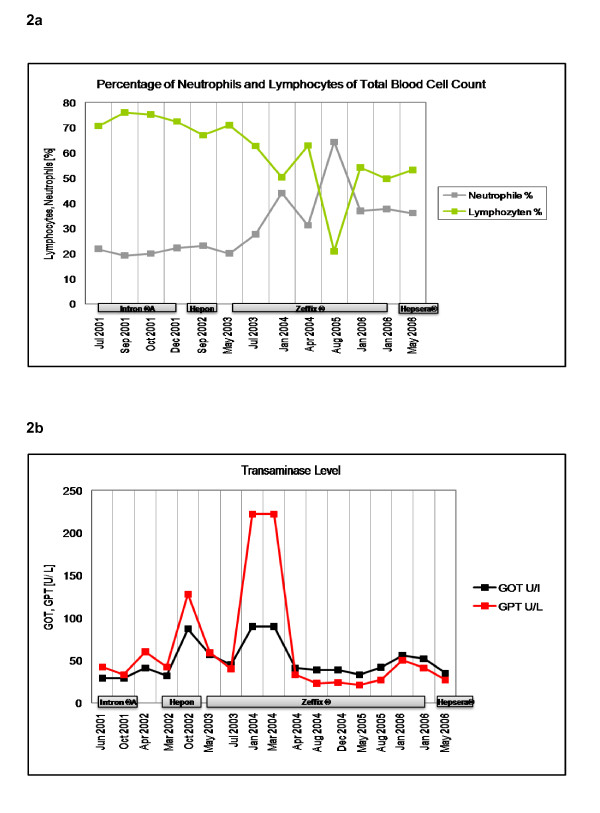
**a. Level of neutrophiles and lymphocytes during CHB treatment with interferon, hepon, lamivudine and adefovir dipivoxil. b**. Level of the transaminases GOT and GPT during CHB patient receiving interferon, hepon, lamivudine and adefovir dipivoxil.

Replacement of Hepon with lamivudine (Zeffix^® ^100 mg per day) resulted in HBV DNA levels below the detection limit of the PCR assay -about 170 molecules of HBV DNA per milliliter, within 12 month. However, the level of transaminases (GOT, GPT) increased drastically (figure 2a). Lamivudine therapy was continued, with a dose adjustment to 40 mg per day and transaminase levels decreased to normal level. After three years of lamivudine treatment resistance developed and the viremia increased again to up to 1.3 × 10^8 ^HBV DNA molecules per mL accompanied by slightly increased GOT levels. This increase of viremia was suspected to be a clinical and virological resistance to lamivudine which could be confirmed by the existence of remaining viral subpopulations with lamivudine resistance mutations (YVDD) (see below). Therapy was switched to adefovir dipivoxil (Hepsera^® ^5 mg per day), which did not respond sufficient and viremia increased further to 7.94 × 10^6 ^molecules per mL.

Throughout the entire observation period, the patient was positive for hepatitis B surface antigen (HBsAg) and had antibodies against hepatitis Bc antigen (anti-HBcAg).

Because lymphocytes are essential inflammation markers in viral infections, the level of neutrophils and lymphocytes were analyzed throughout the observation period as depicted in figure 2b. The percentage of lymphocytes throughout the observation correlates with the level of HBV DNA. During the first 2 years of treatment, only small fluctuations (65-75%) were observed in the amount of lymphocytes. Replacement of Hepon with lamivudine resulted in a decrease in lymphocyte level from 70.9% lymphocytes to 50.2% lymphocytes and, after an intermittent elevation to 62.9% lymphocytes, to 20.8% lymphocytes. When resistance developed, the lymphocyte amount increased again up to 50% lymphocytes. The reference value for the percentage of lymphocytes from total blood cell count lies in a range of 25-40%. The percentage of neutrophils behaved converse to the amount of lymphocytes. During the first 2 years of treatment, only small fluctuations (~20% neutrophils) were observed in amount of lymphocytes. Replacement of Hepon with lamivudine resulted in an increased level of neutrophils from 20% neutrophils to 44% neutrophils and, after an interceptive decrease to 31.2% neutrophils, to 64.3% neutrophils. When lamivudine resistance developed, the percentage of neutrophils decreased again to ~35%. The reference value for the percentage of lymphocytes from total blood cell count lies in a range from 47-72%.

Throughout the observation period, the patient had elevated levels of transaminases (reference value <23U/L). The levels of transaminases during the treatment of CHB with interferon, hepon, lamivudine and adefovir didivoxil are depicted. After application of hepon, GOT increases from 32-57U/L and GPT from 42-128U/L, which significantly decreases again after application of lamivudine to 40U/L GPT and 45U/L GOT. GPT level drastically increase to 222U/L after eight months of lamivudine therapy, which again decreased to 33U/L after adjustment of dosage. Replacement of lamivudine with adefovir dipivoxil again resulted in a slight decrease in both enzymes.

## Gentotypic Analyses

**Figure 3:**
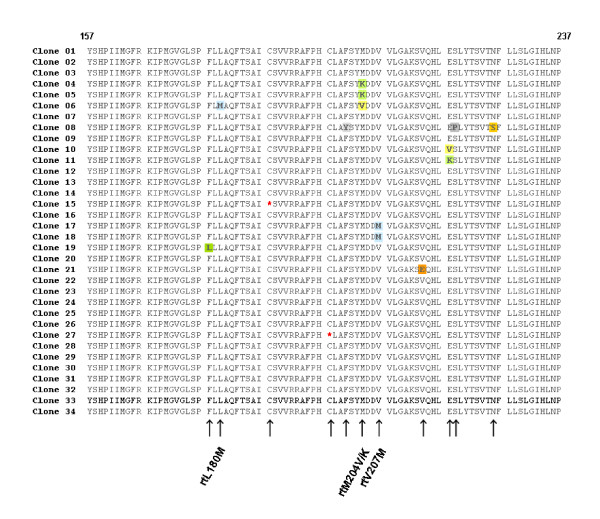
**Alignment of quasispecies analysis**. Sequence of amino acids 157 - 237 in the reverse transcriptase domain of HBV polymerase is shown. The mutations that are associated with lamivudine restistance are at position rt204. The mutations that resulted in a stop codon are highlighted in yellow. A further mutation is located at position rtL180 M The HBV polymerase was amplified from patient material and cloned into TOPO vector, which was transformed into *E. coli*. After purification of plasmid DNA, parts of the *pol *gene were sequenced as described above. Alignment was done using Vector NTi software.

For quasispecies analysis, HBV DNA was isolated from patients' serum after failure of adefovir dipivoxil treatment (06/2007) and parts of the *polymerase *gene of HBV were amplified and cloned into pCR^® ^4-TOPO^® ^vector as previously described [6,7]. A total number of 34 clones were sequenced from both termini and in silico translated into their amino acid sequences (figure 3).

The patient had virus variants containing a mutation resulting in a substitution of leucine to methionine at position rt180, as well as valine to methionine at position rt207. In addition, the patient had a virus variant containing mutations that result in a stop codon (at position rt188 and rt198). The analyses revealed that, beside the above mentioned mutations, also virus variants with mutations in the YMDD motif, namely a substitution of methionine to valine (YVDD) and methionine to lysine (Y**K**DD) at position rt204 were present in patients' serum, which is compatible with lamivudine resistance. Especially this latter mutation rtM204K and the mutation in the direct neighbourhood of the so called YMDD motif, the rtV207 M exchange, may be considered as novel mutations. No further mutations previously associated to antiviral resistance have been detected.

## Discussion and Conclusions

A young male chronically infected with HBV failed several therapy approaches, including INF-α and NRTIs. Quasispecies analyses revealed and proofed the presence of several mutations, including the rtM204V substitution, which is well known to be associated with the selection of lamivudine-resistance virus variants [8-11]. In addition to the known rtM204V substitution, a novel virus variant with a methionine to lysine (YKDD) substitution was found. To the very best of our knowledge this mutation was previously not reported and might also be associated with lamivudine resistance.

Induction of isoleucine or valine in the YMDD motif results in a sterical hindrance, preventing lamivudine from appropriately configuring into the nucleotide binding site of the reverse transcriptase [12], and so the rtM204K substitution may also do. The substitution of lysine to methionine at position rt180 is also connected and approved to contribute to lamivudine resistance and is known to augments the lamivudine resistant in conjunction with rtM204V [11]. Resistance-associated mutations outside the YMDD motiv include amino acid exchanges at the reverse transcriptase codon L180 M (rtL180M) or the rtV207I codon [13]. In this latter report, a novel valine to methionine substitution was present at the above mentioned position rt207. Furthermore, at position rt188 and rt198 nonsense mutations were identified in two clones, which can yield a truncated abbreviated protein often associated with loss of function. As a matter of speculation, such a truncated form of the protein could still be able to bind nucleos(t)ides and their analogues that in turn are not available anymore in a concentration necessary to efficiently suppress viral replication, which besides host factors may have led to the subsequent failure of ADF therapy. Furthermore it cannot be excluded that viral genomes with stop codon mutations may be packaged into Dane particles in liver cells coinfected with wildtype or other viral strains. Surprisingly, despite the well known fact that HBV forms quasispecies, the concept of double infections or even multiinfections of a single liver cell is only rarely taken into account in the analyses of HBV quasispecies. Consequently we assume that the mutations at position rt188 and rt198 may be not artefacts but true mutations, although this hypothesis needs to be tested in further studies.

The selection pressure caused by adefovir can lead to upraise of the mutations rtA181V and rtN236T [14] or rtI233V [6]. None of those substitutions were found in the quasispecies analysis of the patient described. However, as a moderate response to adefovir was observed due to the 2 log drop in viremia the patient's compliance was good and does not explain the stagnation at the high level of viral load.

This might be indicative for further mutations outside the polymerase gene that have not been identified so far. Other options for adefovir treatment failure might be that either the applied prodrugs are not efficiently processed into the active metabolites, or that the drugs are not efficiently delivered to the infected cell by a defective or altered transport mechanism.

Here, as mutations conferring to ADF resistance are missing, it can only be speculated whether mutations in the periphery or a host resistance have contributed to the clinical ADF resistance.

Nevertheless, the case is scholarly for a couple of aspects: On the one hand it shows that there is a definite need for HBV-therapy also in paediatric patients. Guidelines for the treatment of paediatric patients are missing or leaky and clinical studies are rare or completely missing. Chronic HBV infections are rare in fully industrialized countries with more or less intact health care systems but are frequent in countries with emerging markets or in the developing world. In the view of the fact that chronicity of HBV mainly develops if the infection was acquired in the early childhood it must become a major aim to sufficiently treat children that became infected with HBV in order to avoid or reduce chronicity. Thus, consequent and systematic studies for the treatment of children are required.

Furthermore, as shown here by quasispecies analyses, the clinical case presented here is a timely reminder that HBV therapy is not a black and white picture but a highly complicated challenge requiring systematic diagnostic procedures and sophisticated interpretation of results in order to apply the most effective medication schemes.

Finally, the case shows that more educational advertising is required in order to avoid dramatic treatment failures with obscure drugs that may help to give parents some hope that their children could be cured but may lead to severe desperation.

## Competing interests

The authors declare that they have no competing interests.

## Authors' contributions

VS performed the bioinformatical work, analyzed the clinical data, performed parts of the cloning and analyzed the sequencing data. SZ established and performed PCRs and was involved in sequencing. RLT was involved in cloning and sequencing. OS supervised the study, designed the experiments and wrote the manuscript. All authors read and approved the final manuscript.
